# Klf2 as a potential regulator of the signaling Enos/Nhe3/Nherf2 axis in the early adaptive response to MSG-induced renal injury

**DOI:** 10.17912/micropub.biology.002159

**Published:** 2026-06-02

**Authors:** Itzel S Salmon-Cabrales, Luis M Palomino Martínez, Gerardo R Padilla-Rivas, Jose Francisco Islas

**Affiliations:** 1 Biochemistry and Molecular Biology, Autonomous University of Nuevo León, San Nicolás de los Garza, NLE, MX

## Abstract

Oxidative stress-induced renal impairment can result from an overconsumption of monosodium glutamate (MSG). In this context, we analyzed the expression of Krüpple-like factors (KLFs) to identify regulators of ionic homeostasis. Our results suggested that Klf2 is key in regulating the Enos/Nhe3/Nherf2 axis; after identifying putative binding sites with a confidence threshold of over 96%. Suggesting, an early adaptative mechanism in which Klf2 modulates ion transport and vascular function, protecting the kidney from hemodynamic alterations. Thus, Klf2 emerges as an essential regulatory node against nephrotoxicity

**Figure 1. Klf2 as a potential regulator of the Enos/Nhe3/Nherf2 pathway:** **Figure 1.** Klf2 as a potential regulator of the Enos/Nhe3/Nherf2 pathway. (A) Relative mRNA expression of renal *Klf2, Klf4, Klf6, Klf7* , and *Aqp2, Nhe3, Clcnkb2,* and *Kim-1α* . Control group, animals with no MSG treatment. Bars labeled 3, 9, and 15 (days) correspond to distinct groups of rats that received treatment and were euthanized at their respective time points. Data are expressed as fold change (2ˆ ^-∆∆Ct^ ) and are presented as mean ± SEM.Statistical analysis was performed using one-way ANOVA followed by Dunnett’s post-hoc test; *p < 0.05, * *p < 0.01, * * *p < 0.001, * * * *p < 0.0001. (B) Heatmap of Pearson correlation coefficients between *Klfs* and ion channels; specific *R ^2^* values for each correlation are provided within the matrix. (C) Predicted binding sites for the transcription factor *Klf2* within the *Enos/Slc9a3/Slc9a3r2* promoter sequences, as identified by the JASPAR database. The specific profile score threshold for each predicted site is indicated. (D) Hypothetical mechanism of *Klf2* -mediated ionic response regulation following prolonged MSG exposure. MSG exposure promotes an increase in the expression of NHE3 and aquaporin 2 ( *Aqp2* ), suggesting an adaptive response to maintain intracellular sodium levels and osmotic balance, respectively. However, prolonged NHE3 activation can induce cellular damage, hence KLF2 seems to trigger negative feedback to attenuate this effect. To prevent cellular toxicity KLF2 increases the expression of eNOS, promoting the activation of the NO/cGMP signaling cascade, leading to NHE3 inactivation via PKG-dependent phosphorylation; a process facilitated by the scaffolding protein NHERF2.Finally, these findings represent an osmotic coupling mechanism, where the increased sodium reabsorption capacity mediated by the NHE3 axis establishes the gradient necessary for water recovery via aquaporin2 (AQP2). A process attempting to preserve systemic homeostasis in the face of tubular damage ( **Banday, et al., 2011; Li,et al., 2019** ). We recognize that *in silico* binding is a high-probabilistic prediction; therefore, reporter gene assays are necessary to quantify the actual occupancy rate of Klf2 on these promoters, under oxidative stress conditions to validate the functional dependence of this regulatory network. Furthermore, as this is an exploratory study, increasing the sample size in future experiments will be essential to strengthen statistical power and confirm the robustness of these findings.

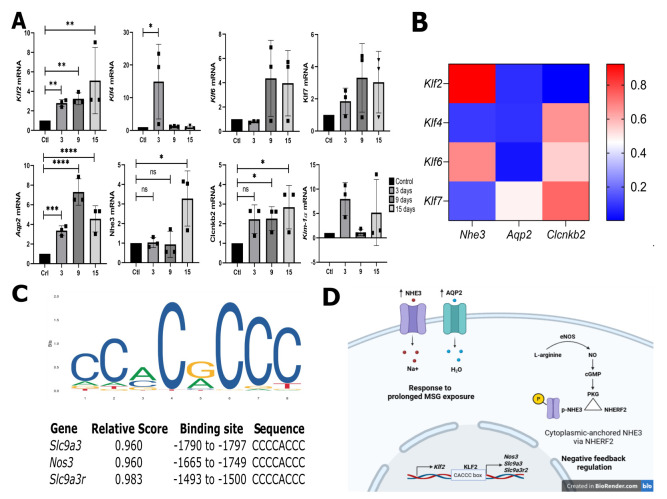



## Description

MSG is a flavor enhancer commonly **used** to prepare ultra-processed foods. At high concentrations, MSG serves as a nephrotoxic agent driven by oxidative stress ( **Airaodion et al., 2020; Makaminan et al., 2025** ). At the molecular level, MSG induces the formation of reactive oxygen species (ROS) that triggers lipid peroxidation, generating cytotoxic secondary metabolites such as malondialdehyde and 4-hydroxynonenal. A process accompanied by an increase in proinflammatory cytokines and a decrease in cellular antioxidant capacity; limiting the synthesis of reduced glutathione ( **Sauganth Paul et al., 2012** ; **Abdou et al., 2025** ). This alteration in cellular redox compromises the kidney's filtration and nutrient reabsorption capacities, both dependent on ion transporters ( **Lee and Han, 2010** ; **Liu et al., 2018** ). In this context, the Sodium-Hydrogen Exchanger 3 (NHE3), encoded by the *Slc9a3* gene, plays a critical role. As an increase in its expression at the renal proximal tubules has been demonstrated as an early adaptive response aimed at regulating volume, homeostasis, and short-term blood pressure ( **Li et al., 2019; Liu et al., 2016; Gonzalez-Vicente et al., 2017** ). The importance of its regulation lies in the fact that sustained activity leads to toxicity due to ionic overload and cellular ATP depletion, as sodium internalization by NHE3 entails a high energy demand ( **Layton, A.T., 2015;** **Albalawy et al., 2024; Cabado et al., 1996** ). To prevent energy decompensation, the cell requires strict homeostatic control. Hence, the scaffolding protein Sodium-Hydrogen Exchanger Regulatory Factor 2 (NHERF2), encoded by the *Slc9a3r2* gene, couples NHE3 with cGMP-dependent protein kinase (PKG), enabling phosphorylation and internalization into cytoplasmic endosomes to induce its inactivation ( **Cha et al., 2005a; Cha and Donowitz, 2008b** ). This negative feedback mechanism, mediated by the eNOS/NO pathway, ensures efficient and controlled sodium reabsorption, protecting the integrity of the tubular epithelium against cytotoxic edema and sustained metabolic stress ( **Chen et al., 2015; Luo et al., 2024; Sarker et al., 2011** ).

Klf2 has been established to regulate eNOS expression, as well as two additional enzymes: argininosuccinate synthetase (ASS) and dimethylarginine dimethylaminohydrolase (DDAH) in the vascular endothelium. Enzymes that promote the convertion of L-arginine to NO, maintain the balance of L-citrulline and L-arginine, and block asymmetric dimethylarginine (ADMA); a competitive inhibitor of eNOS, respectively, thereby ensuring vasodilation, therefore *Klf2,* can act as a potent atheroprotective agent ( **SenBanerjee, et al., 2004; Parmar, et al., 2006** ). However, despite its endothelial relevance, *Klf2* also has a role as a modulator of ion channels to maintain electrolyte balance during the early stages of renal injury. This exploratory study begins to establish Klf2-mediated coordination of ion channel regulation, identifying its relevance in the early adaptive response for maintaining hydro-electrolytic homeostasis under cellular stress conditions.

Male Wistar rats were assigned into four independent groups (n = 3 per group). The control group (Ctl) did not receive MSG treatment. The remaining three distinct groups were subjected to ad libitum oral exposure to 1 g/kg b.w. of MSG for specific durations of 3, 9, and 15 days, and were euthanized at their corresponding time points. During the exposure period, gene expression analysis of renal tissue was performed to evaluate structural transcription factors similar to KLF2 (all subgroup 2), key ion channels ( *Slc9a3* , *Aqp2* , and *Clcnkb2* ), and early injury markers, e.g., *Kim-1α* . Expression results revealed a trend toward increased Kim-1α expression during the first three days following MSG exposure; a sign of acute tubular injury, undetectable in a healthy kidney( **Ichimura, et al., 1998; Han, et al., 2002; Vaidya, Set al., 2010** ). Likewise, a trend toward increased expression of *Klf2* and all ion channels was also observed in MSG exposed groups ( Figure 1A ). Pearson correlation analysis confirmed a robust positive association between *Klf2* and *Slc9a3* (R=0.96922757, R²=0.9394; Figure 1B ). To determine whether this correlation reflected direct transcriptional regulation, an *in-silico* analysis of the proximal promoter region (2000 bp) was performed. We found a single putative Klf2 binding site in the Slc9a3 promoter (positions –1790 to -1797), identifying a site containing the canonical CCCCACCC motif with a relative score of 0.960. It should be noted that this motif was later observed in further searches in both Enos (positions –1493 to -1500) with a relative score of 0.980, and 2 sites in Nherf2 (within positions –1665 to -1749) with a score of 0.960, located very close to the TSS ( Figure 1C ). We should further note that Nhe3 upregulation via eNOS/Klf2 axis is an adaptive mechanism that would require strict control to prevent adverse effects from ionic overload ( **Layton, A.T., 2015;** **Albalawy et al., 2024** ).

The presence of high-affinity binding sites (>96%) in the *Nos3* , *Slc9a3r2* , and *Slc9a3* promoters suggests that, Klf2 acts as a master switch that stabilizes sodium transport function through the eNOS/Nherf2/Nhe3 axis ( Figure 1D ).

In this study, we postulate that Klf2 not only induces *Slc9a3* expression for Na+ reabsorption but also modulates a negative feedback loop for its own inhibition. By inducing *Nos3* , nitric oxide (NO) production increases, activating the NO/cGMP/PKG signaling cascade ( **Arnold, et al., 1977** ; **Gil, et al., 2002** ;). Simultaneously regulating the expression of the Nherf2, Klf2 ensures the coupling of Nhe3 (membrane channel) and PKG; a complex that induces phosphorylation and internalization of Nhe3 into the cytoplasm, thereby limiting excessive sodium entry into the cell.

## Methods

**Animal model**

Male Wistar rats (4-weeks old) were obtained from CIRCULO ADN S.A. de C.V. (Mexico). Animals were maintained under standard environmental conditions with a temperature of 20–24 °C and 27% relative humidity. A 12-hour light/dark cycle was implemented, and rats were provided *ad libitum* access to standard chow and water. Rats were allowed to acclimatize until reaching a body weight of 100 g. The study was conducted in accordance with institutional guidelines and approved by the Research Ethics Committee and the Institutional Animal Care and Use Committee (IACUC) of the School of Medicine, Universidad Autónoma de Nuevo León (Approval No. B125-00004).

**Experimental Model of MSG Exposure**

Male Wistar rats (n = 12) were assigned to four independent groups (n = 3 per group), consisting of an untreated control group (Ctl) and three MSG groups subjected to ad libitum oral exposure (1 g/kg b.w.) for 3, 9, or 15 days prior to euthanasia. For euthanasia, animals were anesthetized with an intraperitoneal injection of Zoletil® 100 (35 mg/kg b.w.; Virbac Mexico, S.A. de C.V.) and Xylazine (10 mg/kg b.w.; Sedaject®, Vedilab S.A. de C.V., Mexico), both diluted in physiological saline to a final volume of 0.3 mL adjusted for body weight. Following sample collection and analysis, euthanasia was confirmed by cervical dislocation.

**Bilateral Nephrectomy**

The peritoneum was exposed through a midline incision extending from the xiphoid process to the pubis. Once the abdominal cavity was opened, the intestines were lateralized to uncover the kidneys. The renal hilum was clamped and severed; the kidney was extracted, and the surrounding adipose tissue was dissected. The kidneys were then preserved at -80 °C for subsequent protein, genetic, and histological analyses. This procedure was repeated for the contralateral kidney.

**RNA Extraction and Reverse Transcription**

Total RNA was extracted from harvested renal tissues using TRIzol® reagent (Invitrogen; Thermo Fisher Scientific, Inc., Waltham, MA, USA) following the manufacturer’s protocol. RNA concentration and purity were assessed using a NanoDrop™ 3000 spectrophotometer (Thermo Fisher Scientific, Inc.) by measuring absorbance at 260 nm and determining the 260/230 and 260/280 ratios. RNA integrity was confirmed via 1% agarose gel electrophoresis, identifying the 28S and 18S ribosomal RNA bands. Subsequently, complementary DNA (cDNA) was synthesized from 250 μg of total RNA using the SuperScript™ VILO™ cDNA Synthesis Kit (Invitrogen; Thermo Fisher Scientific, Inc.) according to the manufacturer’s instructions.

**Quantitative Real-Time PCR (qPCR)**

Target gene expression was analyzed using the SYBR-Green® FAST protocol (Thermo Fisher Scientific, Inc.) on a QuantStudio 7 Real-Time PCR system. Expressions were normalized to *Gapdh* as an internal control. Relative expression levels were calculated using the 2^–∆∆Ct method.

**Sequence Extraction**

Reference genomic sequences for *Rattus norvegicus* were obtained from the UCSC Genome Browser based on mRatBN7.3/2024 genome assembly. For each gene, a 2000 bp window upstream of the TSS was isolated, as defined by the MANE Select reference transcripts.

**Transcription Factor Binding Site Prediction**

Bioinformatic analysis was performed using the JASPAR 2024 database with the Position Weight Matrix MA1515.2 for Klf2. A relative profile score threshold of 95%–98% was applied to minimize false positives and ensure the identification of high-affinity binding sites. This threshold selection follows the recommendations of Wasserman & Sandelin (2004) to optimize the probability of transcription factor occupancy in proximal promoter sequences. Within the JASPAR framework, this threshold serves as a proxy for thermodynamic affinity; thus, the identified proximal promoter sequences of *Nos3* and *Slc9a3r2* exhibit an optimal match score with the position-specific matrix, suggesting a high likelihood of functional protein-DNA interaction (Rauluseviciute et al., 2024; Wasserman & Sandelin, 2004).

**Statistical Analysis**

Data are presented as mean ± standard error of the mean (SEM). Statistical analyses were performed using GraphPad Prism software (version 8.0.2.263, GraphPad Software, San Diego, CA, USA). Given the exploratory nature of the study and the sample size (n=3), a descriptive analysis of expression trends was conducted. Correlations between gene expression levels were evaluated using the Pearson correlation coefficient (R) and the coefficient of determination (R²). A p-value < 0.05 was considered statistically significant for all tests using one-way ANOVA followed by Dunnett’s post-hoc test.

## Reagents

All chemical reagents, including Monosodium Glutamate (MSG), were of analytical grade and purchased from Sigma-Aldrich (St. Louis, MO, USA), unless otherwise specified. Molecular biology reagents, including TRIzol™ and SuperScript™ VILO™ kits, were obtained from Thermo Fisher Scientific, Inc. (Waltham, MA, USA). Primers for *Klf2. Klf4, Klf6, Klf7, Slc9a3* , *Aqp2* , *Clcnkb2* , *Nos3* , and *Kim-1α* were synthesized by T4 Oligo based on sequences retrieved from the NCBI ensembl.
